# Efficacy of liver cancer microwave ablation through ultrasonic image guidance under deep migration feature algorithm

**DOI:** 10.12669/pjms.37.6-WIT.4885

**Published:** 2021

**Authors:** Changkong Ye, Wenyan Zhang, Zijuan Pang, Wei Wang

**Affiliations:** 1 Changkong Ye, Bachelor’s Degrees. Department of Ultrasound, Beihai people’s Hospital, Beihai 536000, China; 2 Wenyan Zhang, Bachelor’s Degrees. Department of Ultrasound, Beihai people’s Hospital, Beihai 536000, China; 3 Zijuan Pang, Master of Medicine. Department of Oncology, Beihai people’s Hospital, Beihai 536000, China; 4 Wei Wang, Bachelor’s Degrees. Department of Oncology, Beihai people’s Hospital, Beihai 536000, China

**Keywords:** Microwave ablation, Liver cancer, Radio frequency ablation, MF, CNN

## Abstract

**Objective:**

To explore the therapeutic effects of ultrasound-guided microwave ablation and radio frequency ablation for liver cancer patients.

**Methods:**

Seventy-eight patients with microwave ablation were rolled into the experimental group and 56 patients with radio frequency ablation were in the control group. This study was conducted from March 1, 2019 to June 30, 2020 in our hospital. Based on Convolutional Neural Networks (CNN) and Migration feature (MF), a new ultrasound image diagnosis algorithm CNNMF was constructed, which was compared with AdaBoost and PCA-BP based on Principal component analysis (PCA) and back propagation (BP), and the accuracy (Acc), specificity (Spe), sensitivity (Sen), and F1 values of the three algorithms were calculated. Then, the CNNMF algorithm was applied to the ultrasonic image diagnosis of the two patients, and the postoperative ablation points, complications and ablation time were recorded.

**Results:**

The Acc (96.31%), Spe (89.07%), Sen (91.26%), and F1 value (0.79%) of the CNNMF algorithm were obviously larger than the AdaBoost and the PCA-BP algorithms (P< 0.05); in contrast with the control group. The number of ablation points in the experimental group was obviously larger, and the ablation time was obviously shorter (P<0.05); the experimental group had one case of liver abscess and two cases of wound pain after surgery, which were both obviously less than the control group (four cases; five cases) (P<0.05)

**Conclusion:**

In contrast with traditional algorithms, the CNNMF algorithm has better diagnostic performance for liver cancer ultrasound images. In contrast with radio frequency ablation, microwave ablation has better ablation effects for liver cancer tumors, and can reduce the incidence of postoperative complications in patients, which is safe and feasible.

## INTRODUCTION

At present, deep learning has been widely used in the field of medical image processing, and the most common deep learning method is convolutional neural network.^1^-^3^ However, it greatly reduces the learning performance of deep learning due to the limitation of the sample size of medical images.^4^-^6^

A new ultrasound image diagnosis algorithm CNNMF was constructed based on CNN and MF, and applied to 78 patients with microwave ablation and 56 patients with radio frequency ablation. By comparing the Acc, Spe, Sen, and F1 values of the CNNMF, AdaBoost, and PCA-BP algorithms and the ablation points and ablation time, the ultrasound-guided microwave ablation and radio frequency ablation were comprehensively evaluated for the treatment effects.

## METHODS

After the IRB approval (dated March 15, 2021), 124 patients with primary liver cancer who entered in our hospital were managed under ultrasound-guided microwave ablation or radio frequency ablation. The study was conducted from March 1, 2019 to June 30, 2020. Seventy-eight cases of microwave ablation were rolled into the experimental group, and 56 patients with radio frequency ablation were included into the control group.

### Inclusion criteria

***I*.** Patients with primary lung cancer confirmed by pathology; ***II*.** Patients with the largest nodule diameter less than 3 cm***; III*.** Patients without contraindications to ultrasound scanning; ***IV*.** Patients with complete clinical history, imaging, etc.

### Exclusion criteria

***I*.** Patients with cholangiocarcinoma and extrahepatic metastasis; ***II*.** Patients with refractory ascites; ***III*.** Patients with unclear consciousness and poor compliance; ***IV*.** Patients who had not received relevant treatments; ***V*.** Patients with mental disorders.

### Ultrasound image based on deep convolution MF

The CNN structure was introduced first.^8^ The input original image was convolved using filters and addable bias, and feature mapping was obtained according to the activation function. Then, the pooling function was adopted to perform dimensionality reduction processing, and a deeper feature map was obtained. After that, all the elements in the feature map were arranged and combined according to a certain order, and output in the fully connected layer.

**Fig.1:**
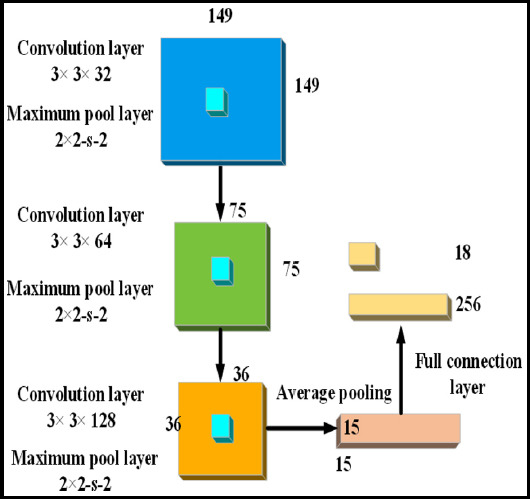
CNN optimized structure.

Using CNN specific network processing can obtain effective learning features, but considering the lack of sufficient image sample data in the field of liver cancer ultrasound, the CNN structure was optimized first, and CNN structures of a three-layer convolutional layer and a pooling layer were designed, known as CNN3. As presented in Fig.1, the size of the input image was 149×149, the feature map size of each layer was {75,35,15}, and the final output neuron was adopted to judge the results.

The size of the convolution kernel used was 3×3, and a smaller convolution kernel can ensure that the depth and effect of the network are improved under the same field of view. Assuming that the size of the input image was 5×5, the parameter set can be expressed as follows.


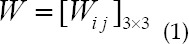


**Fig.2:**
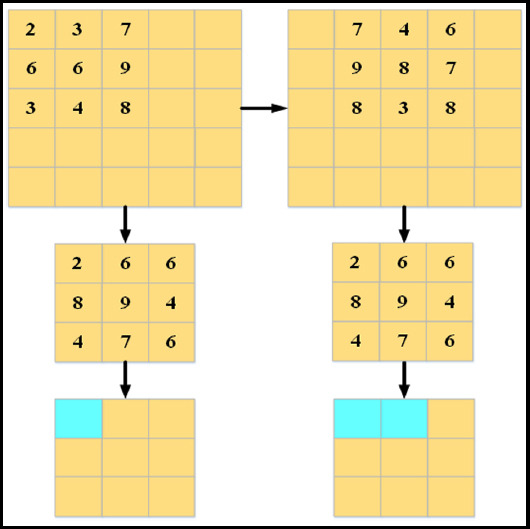
Convolution operation.


In which, W represents the 9 parameter values of the convolution kernel, and i and j represent units in different directions. As shown in Fig.2, the left side represents the process of calculating the output value of the first neuron unit of the feature map, and the right side represents the process of sliding the convolution kernel to the right by one unit length, and the output value of the second neuron in the output map is obtained by convolution operation.

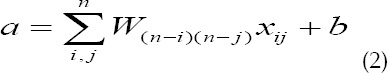


In which, a represents the output value of the neuron, n represents the unit length, x represents the translation coordinate, and b is a constant.

In this study, the largest pooling layer was selected for feature dimensionality reduction. Solving the maximum value of the image blocks separated by every two elements in the pooling layer can reduce the amount of data by 70%.

In addition, it is extremely important to obtain translational invariant features in liver cancer ultrasound images. In this study, the Relu function was selected as the activation function of the model to enhance the nonlinear characteristics of the entire neural network, which was as follows.


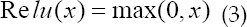


Considering that in the entire CNN, the parameters of the fully connected layer account for about 80%, so the global average pooling (GAP)^9^ operation was adopted for deep feature fusion. Then, the classification of the multi-layer fully connected layer was performed. After the fully connected layer was classified, the Softmax classification function was used as the output layer, which can be expressed as follows.


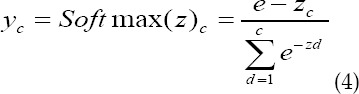


In which, *z_c_* represents the input C-dimensional vector, *y_c_* represents the output C-dimensional vector, 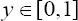, 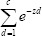 represents a regularized matrix, and 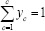. The Softmax function was graphically expressed as follows.


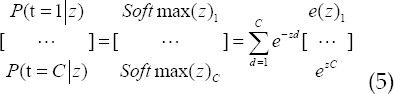


In which, 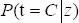 represents the probability of being predicted as class C, which represents the neural layer node, and z represents the predetermined input. MF learning is mainly to find the similarities between source domain data and target domain data at the feature level, to fuse different feature spaces and expand transferable data.^10^ First, the number of fully connected layers and the number of neurons were adjusted to obtain a new network model CNN-B. Then, the feature weight parameters of the trained model CNN-A in the data set were optimized. Finally, fine-tuning the convolution block was carried out. The ultrasound image diagnosis algorithm based on CNN and MF was set as CNNMF.

### Model performance evaluation indicators

The primary lung cancer ultrasound images in The Cancer Genome Atlas (TCGA) database were selected as samples for simulation experiments. The simulation platform was Matlab R2015a, and the operating system was Windows7 64-bit. AdaBoost and PCA-BP based on PCA and BP neural network algorithm were introduced for comparison. The evaluation indicators were Acc, Spe, Sen, and F1 values.


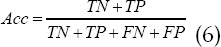



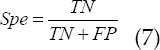



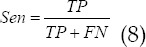



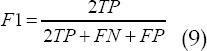


In which, *TP* means true positive, *FP* means false positive, *TN* means true negative, and *FN* means false negative.

### Ultrasound-guided treatment methods

Microwave ablation: the patient was placed in a supine position, and ultrasound-guided location was adopted. The needle insertion site was anesthetized, and a 21G percutaneous transhepatic cholangiography (PTC) needle was placed in the portal vein. Then, 0.5g fluorouracil, 20mg Epirubicin Hydrochloride, and 4mg mitomycin were slowly injected in sequence. After injection, it was washed with saline and the PTC needle was pulled out. The main portal vein was selected during puncture to ensure unobstructed blood flow without reverse flow. After the drug treatment was over, a 15G microwave antenna was pierced into the predetermined site of the tumor in the liver until the strong echo completely covered the tumor. After the microwave antenna entered the predetermined site, 2mg/(kg·time) of propofol needed to be injected through a peripheral vein to make the patient under general anesthesia. Then, microwave ablation was performed. The thermal field superimposition effect was adopted from the inside to the outside for single needle multiple times and multiple needle multiple times for ablation. After the treatment, ultrasound examination was performed again to evaluate the degree of tumor ablation. Radiofrequency ablation: the previous work was the same as above. After anesthesia, the lesion was punctured with an electrode needle. After the electrode was opened, the tumor and the surrounding 0.5cm normal liver tissue were thermally coagulated and necrotic, and multiple needle tract ablations were performed.

### Observation indicators

Basic data (age, height, weight, course of disease, cirrhosis, hypertension, diabetes) were collected for experimental and control groups, the Acc, Spe, Sen, and F1 values of PCA algorithm, BP algorithm, and CNNMF algorithm were compared, and preoperative, intraoperative, and postoperative ultrasound images of patients were obtained. Then, the ablation points and ablation time of the experimental group and the control group were recorded, and postoperative complications (liver abscess, wound pain, biliary fistula) were compared between the experimental group and the control group.

### Statistical Analysis

The data were processed using SPSS19.0, mean ± standard deviation (x(-)± s) was adopted to express the measurement data, and percentage (%) was adopted to express the count data. Paired t-test was adopted to compare the Acc, Spe, Sen, and F1 values of CNNMF, AdaBoost, and PCA-BP algorithms. The number of ablation points, ablation time, and postoperative complications were compared by analysis of variance. P<0.05 meant the difference was obvious.

## RESULTS

### Simulation results analysis

**Fig.3:**
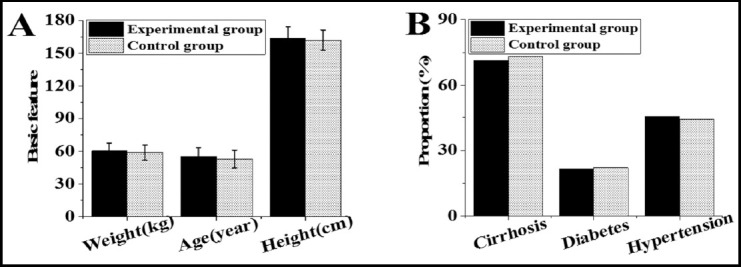
Comparison of basic data.

**Fig.4:**
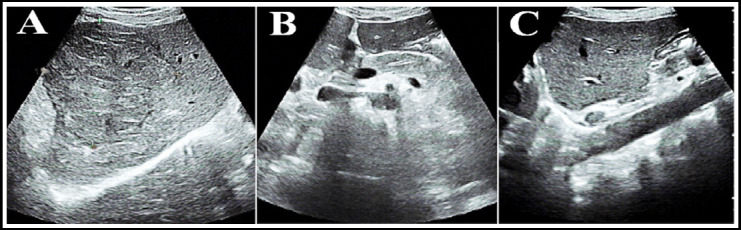
Ultrasound images. ***Note:*** A was an ultrasound image of a female patient (age 68 years old ); B was that of a male patient (age 54 years old); C was that of a male patient (age 57 years old).

As shown in Fig.3, the differences in age, height, weight, liver cirrhosis, hypertension, and diabetes were not obvious (P>0.05). Fig.4 was an ultrasound image of part of the tested patients. Fig.4A showed focal hypoechoic in the liver. The internal echo was relatively uniform and the boundary was clear, and there was an echo halo around the tumor with a diameter of 1.0 cm or more. The anechoic halo around Fig.4B was incomplete or became inconspicuous, there were more tumors, and small sub-nodules appeared around or near the main tumor. Fig.9C showed that the sound compensation behind the tumor and the side sound shadow disappeared, and there was a partial sound wave attenuation behind the larger tumor.

## DISCUSSION

Most patients with primary liver cancer have reached the middle and advanced stages when they are diagnosed, so the liver reserve function is insufficient, and they can’t get the opportunity of surgical resection.^11^ Local thermal ablation therapy provides new treatment opportunities for patients who is no applicable for the surgical resection, and its application in the treatment of patients with advanced liver cancer can inactivate tumors, control tumor growth, and prolong the survival of patients.^12^,^13^ The use of ultrasound-guided thermal ablation therapy can effectively locate the location of the tumor, and provide doctors with information on the size, shape and location of the tumor, so that the doctor can choose a conformal treatment based on the tumor information, thereby improving the effectiveness and safety of the treatment.^14^ Therefore, the effects of different thermal ablation treatments for liver cancer were analyzed and evaluated under ultrasound guidance based on deep learning in this study.

Deep learning requires a lot of data for model training, and it is difficult to obtain a sufficient sample size in the medical field, which limits the development of deep learning.^15^ The use of transfer learning methods can provide ideas for solving this problem. In this study, a new algorithm CNNMF for ultrasound image diagnosis was proposed firstly based on CNN model and migration learning, and it was applied to the ultrasound image processing together with AdaBoost algorithm^16^ and PCA-BP algorithm.^17^ The efficiencies of different algorithms in the identification of lesions in ultrasound images of liver cancer patients were analyzed and compared. The results revealed that compared with AdaBoost and PCA-BP algorithms, the CNNMF algorithm proposed in this study showed higher recognition accuracy, sensitivity, specificity, and F1 value. Accuracy and F1 are often used to evaluate the effect of model classification. The higher the accuracy rate, the better; the higher the recall rate, the better, and the greater the F1 value.^18^-^20^ Based on this, it was proved in this study that the CNNMF algorithm constructed showed higher accuracy and precision in the diagnosis of liver cancer ultrasound images, and can provide a reference for improving the clinical diagnosis rate of liver cancer.

## CONCLUSIONS

In this study, a new ultrasound image diagnosis algorithm CNNMF was constructed based on CNN and MF, which was compared with AdaBoost and PCA-BP algorithms, and applied to ultrasound-guided microwave ablation and radio frequency ablation in the diagnosis of 124 liver cancer patients. It was found that in contrast with the traditional algorithm, the CNNMF algorithm has better diagnostic performance for liver cancer ultrasound images. In contrast with radio frequency ablation, microwave ablation has better ablation effects for liver cancer tumors, higher efficiency, and can reduce the incidence of postoperative complications in patients, which is safe and feasible. However, long-term follow-up is not carried out, and the number termination events is too few, and in future analysis, the sample size of patients should be expanded. In conclusion, the results of this study provide a certain experimental basis for the application of depth feature migration in the diagnosis and treatment of hepatocellular carcinoma ultrasound images.

### Authors’ Contribution:

**CY** conceived the study, literature review, data analysis and drafting of the paper.

**WZ and ZP** helped in design, data collection, article drafting & critical revision.

**WW** is accountable for all aspects of the work in ensuring that questions related to the accuracy or integrity of any part of the work are appropriately investigated and resolved.
